# Evaluation of preoperative anxiety and fear of anesthesia using APAIS score

**DOI:** 10.1186/s40001-018-0339-4

**Published:** 2018-09-11

**Authors:** Fatma Celik, Ipek S. Edipoglu

**Affiliations:** 1 Department of Anesthesiology and Intensive Care Medicine, Ahi Evran University Medical Faculty, Kirşehir, Turkey; 2 Department of Anesthesiology, Suleymaniye Obstetrics & Pediatrics Training and Research Hospital, Istanbul, Turkey

**Keywords:** APAIS, Anxiety, Fear of anesthesia

## Abstract

**Background:**

Preoperative anxiety is one of the most important problems for the patients, because it causes emotional and psychiatric problems as well as physical problems. It is crucial to detect the patient’s existing anxiety to assist patients. Our primary aim in this study is to investigate how the patient’s age, gender, the operation, surgical briefing, type of anesthesia recommended for the operation ahead, and patient’s prior anesthesia experience affect the patient’s anxieties. Our secondary aim is to reveal the causes of the patient’s anxieties regarding anesthesia.

**Methods:**

Our study was conducted as a prospective cohort study between May 2016–2017. Interviews with the patients were performed in the anesthesia clinic for preoperative examination. For the study, The Amsterdam Preoperative Anxiety and Information Scale (APAIS) has been used. The answers were evaluated in two scales: the anxiety score and the desire for information score. Answers to the statements were evaluated with Likert Scale. In addition, our patients were asked whether they had received prior anesthesia, if so, the type of anesthesia, whether they received surgical briefing and anesthetic method we recommended. We also asked our patients about the cause of their anxiety regarding the anesthesia.

**Results:**

A total of 637 patients were recruited to the study, after excluding the patients who do not meet the criteria for inclusion, and 499 patients were included. Between the age and desire for information sub-scores, a negative significant correlation was detected (*r*: − 0.241; *p* = 0.001). We found that the scores of graduates of university and higher were statistically significant than the primary school graduates (*p* = 0.003) and secondary school graduates (*p* = 0.034). Anxiety sub-scores of the patients who underwent general anesthesia were found to be significantly higher than the patients who underwent regional anesthesia (*p* = 0.029). Anxiety sub-scores of females were found to be significantly higher than the males (*p* = 0.001).

**Conclusions:**

We think that being aware of the patients’ anxiety and finding appropriate approaches for their anxieties can be valuable. APAIS is an effective method to measure patient anxiety and it might be beneficial to use during preoperative visits. Patient satisfaction and superior outcomes can be achieved in this way.

*Trial registration* ISRCTN43960422. Registered 19/02/2018—Retrospectively registered. http://www.isrctn.com/ISRCTN43960422

## Introduction

Preoperative anxiety is one of the most important problems for the patients, because it causes emotional and psychiatric problems as well as physical problems [1]. Anxiety is particularly important, because it has the potential to affect all aspects of anesthesia such as preoperative visit, induction, perioperative, and recovery periods [2, 3]. Perioperative anxiety is found to be correlated with increased autonomic fluctuations and increased requirement of anesthetic, elevated incidence of nausea and vomiting, and augmented pain during postoperative period [4–6]. As a result of these complications, it was reported that recovery period and the length of hospital stay were extended [4]. High levels of anxiety were seen in many patients during the preoperative period and all patients had different levels of anxiety. The exact etiology of anxiety can be due to anesthesia, surgery, and several other different reasons [7–9]. Thus, it is crucial to detect the patient’s existing anxiety to assist patients. Many different approaches have been reported on this subject, but some of the methods are not practical to use and can be time consuming during the preoperative preparatory period due to non-specific questions [10]. In 1996, Amsterdam Preoperative Anxiety and Information Scale (APAIS) has been developed as a practical method [10, 11]. Preoperative anxieties of the patients scheduled for surgery can be evaluated using APAIS.

Our primary aim in this study is to investigate how the patient’s age, gender, the operation, surgical briefing, type of anesthesia recommended for the operation ahead, and patient’s prior anesthesia experience affect the patient’s anxieties regarding anesthesia and surgery. Our secondary aim is to reveal the causes of the patient’s anxieties regarding anesthesia if there are any.

## Materials

**Fig. 1:**
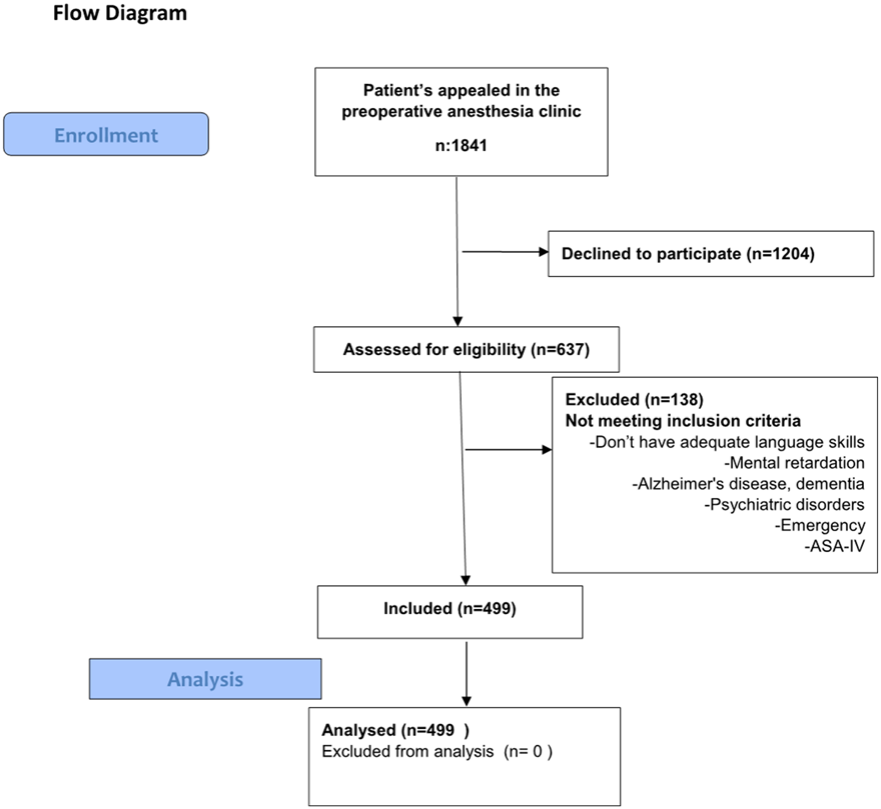
Flow chart

This study was conducted as a prospective cohort study and was performed during the 1-year period between May 2016 and 2017. The study was performed on patients admitting to the anesthesia outpatient clinic for preoperative examination in Ahi Evran University’s faculty of medicine. ASA I–III, adult patients who are scheduled to undergo elective surgery in our hospital and have adequate language skills for the interview were included in our study. Pediatric patients, patients who did not give consent, patients with mental retardation, Alzheimer’s disease, dementia or psychiatric disorders, emergency, and ASA-IV patients were excluded from the study. Selection of patients was described in the flow diagram (Fig. 1). A written informed consent has been obtained prior to the interviews.

Interviews with the patients were performed when they came to anesthesia clinic for preoperative examination, under the supervision of a specialist anesthetist. The questioning was conducted before the premedication visit in the waiting room. The interviewer was a nurse who was totally blinded to our study. For the study, The Amsterdam Preoperative Anxiety and Information Scale (APAIS), which comprises six statements, has been used. The answers were evaluated in two scales: the anxiety score and the desire for information score. Anxiety score was obtained by calculating the total scores assigned to the expressions “ I am worried about the anesthesic”, “The anesthesic is on my mind continually”, “I am worried about the procedure”, “The procedure is on my mind continually”, to measure the patient’s level of anxiety regarding the anesthesia and surgery. Desire for information score is obtained by calculating the total scores assigned to the expressions “I would like to know as much as possible about the anesthesic “ and “I would like to know as much as possible about the procedure “to measure the patient’s level of desire for information regarding the anesthesia and surgery. Higher scores indicate higher levels of anxiety and desire for information. Answers to the statements were evaluated with Likert Scale. (1-Not at all…,5-Extremely). In addition, our patients were asked whether they had received anesthesia due to any reason, if so, the type of anesthesia, and whether they received surgical briefing. We also recorded which anesthetic method we recommended to the patient for the surgery they will undergo in the preoperative anesthesia clinic.

Then, we asked our patients about the cause of their anxiety regarding the anesthesia if there are any:

1. fear of death;
2. fear of waking up in middle of the surgery;
3. postoperative pain;
4. postoperative nausea, vomiting;
5. become permanently disabled;
6. experience of anesthesiologist;
7. fear of needle, intervention;
8. feeling pain during the surgery;
9. other.

The patients were asked to select more than one cause if it is the case.

### Statistical analysis

For the statistical analysis, NCSS (Number Cruncher Statistical System) 2007 (Kaysville, Utah, USA) program was used. When evaluating the study data, in addition to the descriptive statistical methods (mean, standard deviation, median, frequency, ratio, minimum, and maximum), when comparing quantitative data, for the variables that do not have normal distribution, Mann–Whitney *U* test was used to compare two groups. Kruskal–Wallis test was used to compare three or more groups and Dunn–Bonferroni as a post hoc test. To evaluate the correlation between the parameters, Spearman’s correlation analysis was used. *p* value smaller than < 0.05 was considered significant.

## Results

The study was carried out in university hospital, within the period of 1 year. A total of 637 patients were recruited to the study, but after excluding the patients who do not meet the criteria for inclusion, 499 patients were included. Of these 499 patients, 47.9% (*n* = 239) were female, and 52.1% (*n* = 260) were male. The ages of the patients ranged between 18 and 92, and the mean age was 42.79 ± 14.75 years. In Table 1, demographic characteristics of the patients were presented, and most of the patients (45.2%, *n* = 225) were primary school graduates, which was the lowest level of education. In contrast, the ratio of the patients with the highest level of education, PhD, was 1.8% (*n* = 9).

**Table 1:** Demographic data

Age (years)	
Min–max (median)	18–92 (43)
Mean ± SD	42.79 ± 14.75
Gender, *n* (%)	
Female	239 (47.9)
Male	260 (52.1)
Education, *n* (%)	
Primary school	225 (45.2)
Secondary school	60 (12.0)
High school	116 (23.2)
University	89 (17.8)
Doctorate	9 (1.8)

*SD* standard deviation, *n* number

It was seen that the patients most frequently undergo septorhinoplasty, with 17.2% (*n* = 86). Due to the variety of operations performed in our hospital, we classified a significant portion (41.9%, *n* = 209) of the operations that our patients undergo under the title “other”. It was found that 43.9% of our patients (*n* = 219) were operated under regional anesthesia. 62.9% (*n* = 314) of the cases had anesthesia experience, 72.6% (*n* = 228) of these cases had general anesthesia, 14.3% (*n* = 45) had regional anesthesia, and 13.1% (*n* = 41) had both general and regional anesthesia (Table 2). Only 59.7% of the cases (*n* = 298) received surgical briefing.

**Table 2:** Features of surgery and anesthesia distributions

	*n*	%
Operation type		
Septorhinoplasty	86	17.2
Cholecystectomy	60	12.1
Inguinal hernia	55	11.0
Ureteroscopy	42	8.4
Endoscopy	26	5.2
Pilonidal sinus	21	4.2
Others	209	41.9
Anesthesia type		
General anesthesia	280	56.1
Regional anesthesia	219	43.9
History of anesthesia		
No	185	37.1
Yes	314	62.9
History of general anesthesia	228	72.6
History of regional anesthesia	45	14.3
History of both general and regional anesthesia	41	13.1
Surgical consent		
No	201	40.3
Yes	298	59.7

*SD* standard deviation, *n* number

Total APAIS score of the cases ranged between 6 and 30, and the mean was 14.50 ± 4.77. Anxiety scores of our patients ranged between 4 and 20, and the mean was 6.75 ± 3.73. The scores for the desire for information ranged between 2 and 10, and the mean was 7.75 ± 2.03. The distribution regarding “The Amsterdam Preoperative Anxiety and Information Scale (APAIS)” of the cases is given in Table 3.

**Table 3:** APAIS Scale distributions

	Not at all		Slightly		Moderately		Very		Extremely	
	*n*	%	*n*	%	*n*	%	*n*	%	*n*	%
1. I am worried about the anesthesic	278	55.8	118	23.6	28	5.6	45	9.0	30	6.0
2. The anesthesic is on my mind continually	395	79.2	58	11.6	16	3.2	21	4.2	9	1.8
3. I would like to know as much as possible about the anesthesic	28	5.6	12	2.4	95	19.0	224	44.9	140	28.1
4. Am worried about the procedure	261	52.3	99	19.8	55	11.1	48	9.6	36	7.2
5. The procedure is on my mind continually	362	72.5	73	14.7	26	5.2	21	4.2	17	3.4
6. I would like to know as much as possible about the procedure	34	6.8	17	3.4	76	15.2	221	44.3	151	30.3

**Fig. 2:**
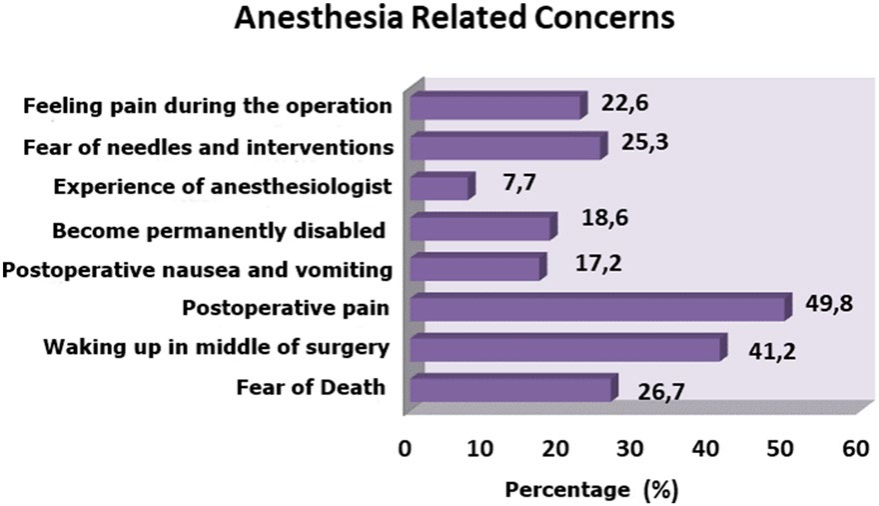
Anesthesia-related concerns

After the interviews, it was found that of the 221 cases (44.3%) with anxieties regarding the anesthesia. Most of them, 41.2% (*n* = 91) were anxious about waking up during surgery, 49.8% (*n* = 110), and were anxious about postoperative pain. We demonstrated the other reasons of concerns in Fig. 2.

No statistically significant correlation was detected between the anxiety sub-scores in terms of age (*p* > 0.05). Between the age and desire for information sub-scores, a negative correlation statistically significant by 24.1% was detected (*r*: − 0.241; *p* = 0.001). Between the age and total scores, a negative correlation statistically significant by 18.1% was detected (*r*: − 0.181; *p* = 0.001) (Table 4).

**Table 4:** Association of demographic data and APAIS sub-scores and total score

	*n*	APAIS scale		
		Anxiety score	Information wish score	Total score
		Min–max (median) Mean ± SD	Min–max (median) Mean ± SD	Min–max (median) Mean ± SD
Age (years)				
*r*	499	− 0.092	− 0.241	− 0.181
*p*		0.059	0.001**	0.001**
Gender				
Female	239	4–20 (6)	2–10 (8)	6–30 (14)
		7.77 ± 4.24	7.82 ± 1.96	15.58 ± 5.27
Male	260	4–20 (4)	2–10 (8)	6–29 (13)
		5.81 ± 2.90	7.69 ± 2.09	13.50 ± 4.03
*p* ^a^		0.001**	0.461	0.001**
Education				
Primary school	225	4–20 (5)	2–10 (8)	6–30 (13)
		6.66 ± 3.80	7.25 ± 2.13	13.91 ± 4.98
Secondary school	60	4–18 (5)	2–10 (8)	6–26 (13)
		6.03 ± 3.28	7.75 ± 1.97	13.78 ± 4.20
High school	116	4–20 (6)	2–10 (8)	6–30 (14)
		6.87 ± 3.53	8.09 ± 1.72	14.96 ± 4.35
University and above	98	4–20 (6)	2–10 (9)	6–29 (14)
		7.24 ± 4.00	8.51 ± 1.88	15.76 ± 4.85
*p*^b^		0.188	0.001**	0.001**

^a^Mann–Whitney *U* Test; ^b^ Kruskall–Wallis Test; *p < 0.05; **p < 0.01; *r* Spearman’s correlation

*SD* standard deviation, *n* number

**Fig. 3:**
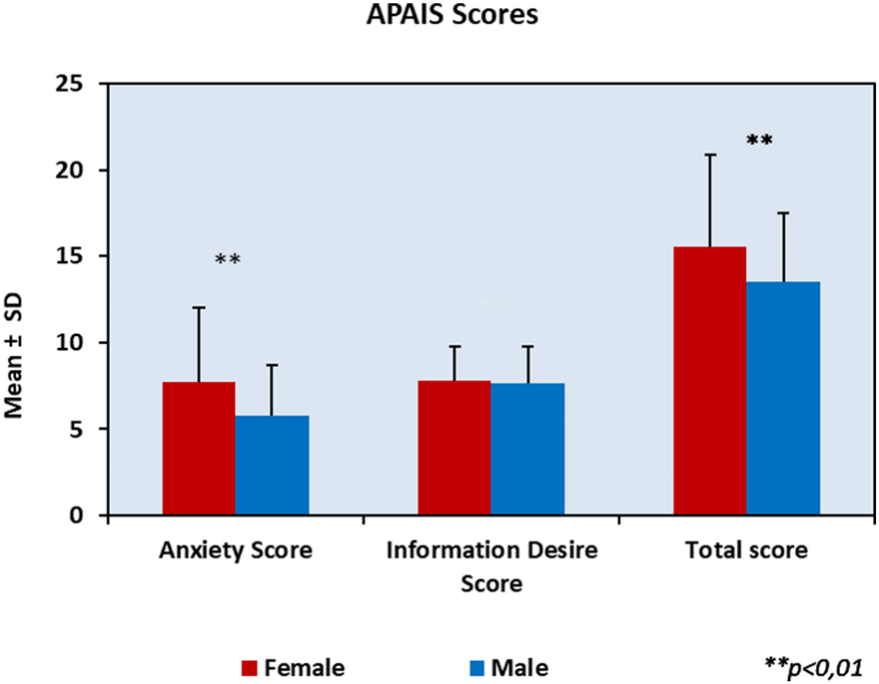
APAIS sub-scores, total score, and their association with gender

Anxiety sub-scores of females were found to be statistically significantly higher than the males (*p* = 0.001). No statistically significant difference was detected between the desires for information sub-scores in terms of gender (*p* > 0.05). Total scores of female patients were statistically significantly higher than the scores of the male patients (*p* = 0.001) (Fig. 3).

**Fig. 4:**
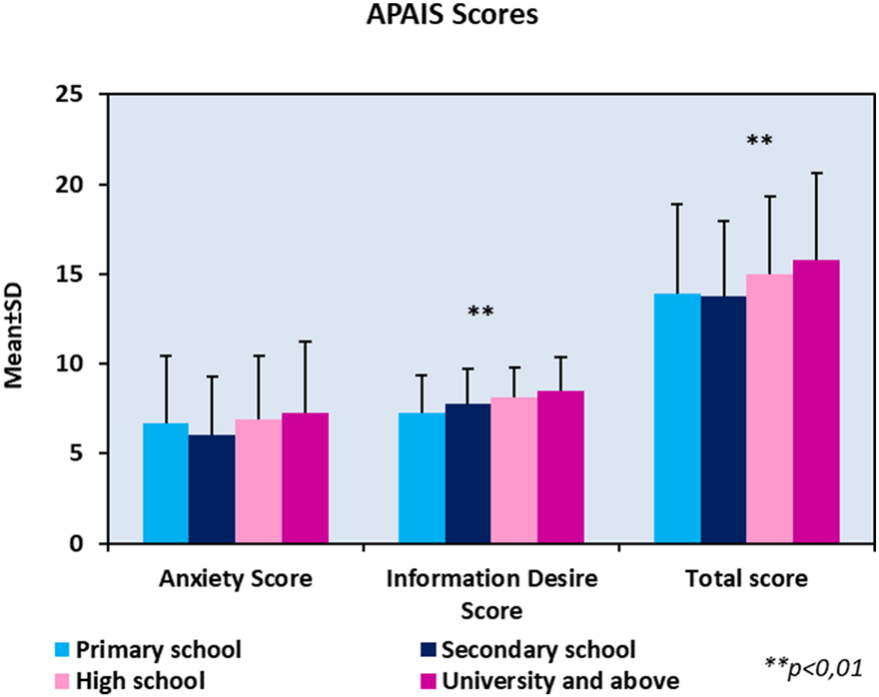
APAIS sub-scores, total score, and their association with level of education

No statistically significant difference was detected between the anxiety scores in terms of education-level sub-scores (*p* > 0.05). Statistically significant difference was detected between the scores of desires for information sub-scores in terms of education level (*p* = 0.001). According to the dual comparisons performed to detect the group causing the significant difference, it was found that the scores of primary school graduates are significantly lower than the scores of high school graduates (*p* = 0.003) and graduates of university and higher (*p* = 0.001) (*p* < 0.01). The scores of secondary school graduates were significantly lower than the graduates of university and higher (*p* = 0.031). Statistically significant difference was detected between the total scores in terms of education level (*p* = 0.001). According to the dual comparisons performed to detect the group causing the significant difference, it was found that the scores of graduates of university and higher were statistically significant than the primary school graduates (*p* = 0.003) and secondary school graduates (*p* = 0.034) (Fig. 4).

Anxiety sub-scores of the patients who underwent general anesthesia were found to be statistically significantly higher than the patients who underwent regional anesthesia (*p* = 0.029) (Table 5).

**Table 5:** Association of anesthesia and surgical features and APAIS sub-scores and total scores

	*n*	APAIS scale		
		Anxiety score	Information desire score	Total score
		Min–max (median) Mean ± SD	Min–max (median) Mean ± SD	Min–max (median) Mean ± SD
Anesthesia type				
General anesthesia	280	4–20 (6)	2–10 (8)	6–30 (14)
		7.01 ± 3.84	7.81 ± 2.14	14.82 ± 4.98
Regional anesthesia	219	4–19 (5)	2–10 (8)	6–29 (13)
		6.42 ± 3.56	7.67 ± 1.88	14.09 ± 4.48
*p*^a^		0.029*	0.112	0.045*
History of anesthesia				
No	185	4–19 (5)	2–10 (8)	6–29 (14)
		6.76 ± 3.60	8.06 ± 1.96	14.82 ± 4.76
Yes	314	4–20 (5)	2–10 (8)	6–30 (13)
		6.74 ± 3.81	7.57 ± 2.05	14.31 ± 4.78
*p*^a^		0.802	0.003**	0.123
Type of anesthesia history (n = 314)				
General anesthesia	228	4–20 (5)	2–10 (8)	6–30 (13)
		6.53 ± 3.67	7.57 ± 2.04	14.10 ± 4.77
Regional anesthesia	45	4–20 (5)	2–10 (8)	7–29 (13)
		7.11 ± 4.42	7.58 ± 2.15	14.69 ± 5.09
Both general and regional anesthesia	41	4–18 (6)	2–10 (8)	6–26 (14)
		7.54 ± 3.80	7.54 ± 2.01	15.07 ± 4.54
*p*^b^		0.135	0.977	0.427
Surgical consent				
No	201	4–20 (5)	2–10 (8)	6–29 (13)
		6.92 ± 3.89	7.64 ± 1.91	14.56 ± 4.91
Yes	298	4–20 (5)	2–10 (8)	6–30 (14)
		6.63 ± 3.62	7.83 ± 2.10	14.46 ± 4.69
*p*^a^		0.442	0.119	0.977

^a^Mann–Whitney *U* Test; ^b^Kruskall–Wallis Test; *p < 0.05; **p < 0.01

*SD* standard deviation, *n* number

No statistically significant difference was detected between the desire for information scores in terms of the type of anesthesia (*p* > 0.05). Total scores of the patients who underwent general anesthesia were statistically significantly higher than the patients who underwent regional anesthesia (*p* = 0.045). No statistically significant difference was detected between the anxiety sub-scores in terms of anesthesia experience (*p* > 0.05). Desire for information sub-scores of the patients with no experience of anesthesia was statistically significantly higher than the patients with experience (*p* = 0.003). No statistically significant difference was detected between the total scores in terms of anesthesia experience (*p* > 0.05).

Anxiety sub-scores (*p* = 0.001), desire for information sub-scores (*p* = 0.001), and total scores (*p* = 0.001) of the patients anxious regarding anesthesia were found to be statistically significantly higher than the patients who do not feel anxiety (Table 6). Of the patients who had fear of death, anxiety subdimension scores (*p* = 0.001) and total scores (*p* = 0.001) were found to be statistically significantly higher than the patients who do not have fear of death. Of the patients who have the fear of waking up during the surgery, anxiety sub-scores (*p* = 0.013) and total scores (*p* = 0.009) were statistically significantly higher than the patients who do not have the fear of waking up. While there was no statistically significant difference in anxiety sub-scores and total scores in terms of postoperative anxiety (*p* > 0.05), desire for information sub-scores of the patients with postoperative anxiety was significantly higher than the patients who do not have the anxiety (*p* = 0.031). It is striking that the patients who have anxiety regarding nausea and vomiting did not have high anxiety sub and total scores. Patients who have fear of becoming disabled have a significantly higher anxiety sub-scores (*p* = 0.001) and total scores (*p* = 0.001) than the patients who do not have this fear. While no statistically significant difference was detected between the desire for information subdimension scores in terms of fear of becoming disabled, it is noteworthy that the patients with this fear have high scores (*p* > 0.05). The patients who have anxiety regarding the inadequacy of the anesthetist have statistically significantly higher anxiety sub-scores (*p* = 0.001), desire for information sub-scores (*p* = 0.046) and total scores (*p* = 0.002) than those who do not have this anxiety. While there is no statistically significant difference between the desire for information sub-scores and between the total scores in terms of the fear of needle or intervention (*p* > 0.05), anxiety sub-scores of the patients with this fear were statistically significantly higher than the patients who do not have this fear (*p* = 0.028). Anxiety sub-scores (*p* = 0.004) and total scores (*p* = 0.003) of the patients who have anxiety of feeling pain during surgery are statistically significantly higher than the patients who do not have this anxiety.

**Table 6:** Association of anesthesia-related concerns and APAIS sub-scores and total scores

	*n*	APAIS Skalasi		
		Anxiety score	Information desire score	Total score
		Min–max (median) Mean ± SD	Min–max (median) Mean ± SD	Min–max (median) Mean ± SD
Anesthesia-related concerns				
No	278	4–14 (4)	2–10 (8)	6–22 (12)
		4.60 ± 1.39	7.35 ± 2.06	11.95 ± 2.61
Yes	221	5–20 (8)	2–10 (8)	7–30 (17)
		9.45 ± 3.98	8.26 ± 1.87	17.71 ± 4.95
*p*^a^		0.001**	0.001**	0.001**
The reasons of anesthesia-related concerns (*n* = 221)				
Fear of death				
No	162	5–20 (8)	2–10 (8)	7–30 (16)
		8.83 ± 3.60	8.19 ± 1.96	17.01 ± 4.70
Yes	59	5–20 (10)	2–10 (9)	12–30 (19)
		11.15 ± 4.48	8.47 ± 1.59	19.63 ± 5.14
*p*^a^		0.001**	0.519	0.001**
Waking up in middle of surgery				
No	130	5–19 (8)	2–10 (8)	7–29 (16)
		8.81 ± 3.53	8.09 ± 2.05	16.9 ± 4.70
Yes	91	5–20 (9)	2–10 (9)	11–30 (18)
		10.36 ± 4.42	8.51 ± 1.56	18.87 ± 5.08
*p*^a^		0.013*	0.251	0.009**
Postoperative pain				
No	111	5–20 (8)	2–10 (8)	8–30 (16)
		9.35 ± 3.96	8.07 ± 1.82	17.42 ± 4.96
Yes	110	5–20 (8)	2–10 (9)	7–30 (17)
		9.55 ± 4.02	8.45 ± 1.91	18.00 ± 4.94
*p*^a^		0.711	0.031*	0.225
Postoperative nausea and vomiting				
No	183	5–20 (8)	2–10 (8)	7–30 (16)
		9.20 ± 3.84	8.19 ± 1.92	17.38 ± 4.80
Yes	38	5–20 (10)	3–10 (9)	11–30 (19)
		10.66 ± 4.46	8.63 ± 1.57	19.29 ± 5.40
*p*^a^		0.057	0.204	0.060
Become permanently disabled				
No	180	5–20 (8)	2–10 (8)	7–29 (16)
		8.72 ± 3.54	8.18 ± 1.88	16.89 ± 4.48
Yes	41	6–20 (12)	3–10 (9)	11–30 (22)
		12.66 ± 4.26	8.63 ± 1.81	21.29 ± 5.33
*p*^a^		0.001**	0.058	0.001**
Experience of anesthesiologist				
No	204	5–20 (8)	2–10 (8)	7–29 (16)
		9.16 ± 3.78	8.22 ± 1.85	17.37 ± 4.73
Yes	17	6–20 (12)	3–10 (10)	11–30 (22)
		12.94 ± 4.8	8.82 ± 2.01	21.76 ± 5.77
*p*^a^		0.001**	0.046*	0.002**
Fear of needles and interventions				
No	165	5–20 (8)	2–10 (9)	7–30 (16)
		9.13 ± 3.89	8.42 ± 1.69	17.55 ± 4.67
Yes	56	5–20 (10)	2–10 (8)	8–30 (18)
		10.38 ± 4.13	7.80 ± 2.28	18.18 ± 5.70
		0.028*	0.139	0.600
Feeling pain during the operation				
No	171	5–20 (8)	2–10 (8)	7–30 (16)
		9.06 ± 3.85	8.25 ± 1.80	17.31 ± 4.82
Yes	50	5–20 (10)	2–10 (9)	11–30 (18.5)
		10.76 ± 4.19	8.32 ± 2.10	19.08 ± 5.17
*p*^a^		0.004**	0.393	0.003**

^a^Mann–Whitney *U* Test; *SD* standard deviation, *n* number

*p < 0.05

**p < 0.01

## Discussion

In our study, we aimed to evaluate patient anxiety using APAIS scale and to reveal the causes of fear in patients with anxiety regarding anesthesia. APAIS is a suitable tool to measure anxiety score, as its anxiety and desire for information sub-scores reveal important information on anesthesia and surgery [12]. At the same time, as it is planned with far-reaching answers, quickly performed and easy to understand, APAIS is a scale widely well known by patients and doctors [12]. These are the reasons why we preferred APAIS over State–Trait Anxiety Inventory, (STAI).

In this study, we found a negative correlation between the age and desire for information. We found that as our patients get older, their desire for information decreases (*r*: − 0.241; *p* = 0.001). Similar to our study, a comprehensive study which investigated all patient groups found that older patients have less anxiety [13]. Taşdemir et al. [7] also reported that elderly patients have lower anxiety scores in preoperative period. In another study, it was proposed that the reason behind this is the possible fatalistic viewpoint of the elderly patients and the efficient use of the communication instruments by the youngsters and the subsequent high probability of accessing bad outcomes/experiences on health [14]. There are studies in the literature, which report different results, on the correlation between anxiety and age [13].

Analysis of anxiety scores showed that females have higher anxiety scores than males. In the literature, there are many studies that corroborate the results of our study [13, 15]. The authors think that this is because traditionally, males cannot easily express their weaknesses [15]. However, there are studies that report different results on the correlation between gender and anxiety [10]. Experimental studies suggested that the fluctuations in estrogen and progesterone levels can be the source of mood and anxiety disorders and the cause of the difference in female patients [16].

We found that the desire for information scores increases statistically as the level of education increases. In the literature, there are conflicting results on this subject. While some of them reported an increase in the anxiety with education, some reported a decrease [17]. In the study that included 592 patients, Cauomo et al. [18] found that anxiety scores increased with increased education levels. They suggested that the reason behind this was that educated patients can express themselves better and they had higher awareness on anesthesia and surgery [18].

We found that patients scheduled for general anesthesia have statistically higher anxiety scores than patients scheduled for regional anesthesia. The potential reason behind this can be that with regional anesthesia, patients think that they will be awake during the operation and thus will have consciousness of the surroundings. Similarly, general anesthesia can increase the patient anxiety, as the patients think that they will not have control and will be at the mercy of the healthcare staff during the operation.

As expected, we found that the patients with no previous experience of anesthesia have higher desire for information scores. Similar to our study, there are studies reporting that the previous surgery experience decreases the anxiety levels [18, 19]. The correlation between previous anesthesia experience and anxiety was shown in the literature [1]. Differences between the studies can be due to the positive or negative personal experiences of the patients.

Patients who had anxiety of anesthesia most frequently had the fear of postsurgical pain (*n* = 110), followed by waking up during the surgery (*n* = 91). We found that anxiety of death came only the third (*n* = 59). Similar to our study, a recent survey study showed that the fear of pain was the most important cause of anxiety [20]. In another study with 800 patients by Sheved et al. [13], similar results were reported on fear of pain. The survey studies by Burkle et al. [17] showed that the fear of death was the biggest cause of anxiety. They also reported that the fear of death decreases with increasing age. The difference between the aforementioned studies and our study is that the questionnaires in those studies do not include “pain during perioperative period” option included in our study. As expected, fear of needle or intervention ranked the fourth, with 25.3% of the patients. We found that 22.6% of our patients were anxious about feeling pain during the surgery, 18.6% were anxious about becoming disabled, and 17.2% were anxious about postoperative nausea and vomiting. The reason that caused the least amount of anxiety was the inadequacy of the anesthetist (*n* = 17). Information on the factors that affect anxiety may reduce preoperative anxiety [21]. Studies on anesthesia-induced anxiety report various results that are close to each other. In a study with 100 patients by Matthias et al., it was found that “awareness” during anxiety is the most important cause of anxiety. It was followed by the surgical outcome, postoperative pain, waiting for the surgery, and being at the mercy of the healthcare personnel [21]. In another study, the authors reported that the causes of anxiety are, respectively, waiting for the surgery, being at the mercy of the healthcare personnel, surgical outcome, and awareness [22]. We think that these small differences between the studies may be caused by some sociological differences between the countries.

In our study, using APAIS, we also investigated the causes of anxiety regarding anesthesia. In our study, both the anxiety scores and desire for information scores of the patients who felt anxious regarding anesthesia were statistically significantly higher than the patients who did not feel anxious. Patients who had fear of death had statistically significantly higher anxiety scores than the patients who did not have this fear. Patients who had fear of waking up during surgery had statistically significantly higher anxiety scores than those who did not have this fear. Desire for information scores was significantly higher in patients anxious about postoperative pain than the patients who did not have this anxiety. Anxiety scores of patients who had fear of becoming disabled were statistically significantly higher than those who did not have this fear. Anxiety scores and desire for information sub-scores of patients anxious about the inadequacy of the anesthetist were statistically significantly higher than the patients who did not have this anxiety. Anxiety sub-scores of patients who had fear of needle or intervention were significantly higher than patients who did not have this fear. The anxiety subscale scores of the patients who had anxiety of waking up during the surgery were statistically significantly higher than the patients who did not have this anxiety. Among all these causes, we failed to detect a correlation between anxiety and nausea and vomiting. As a result of our findings, we demonstrated the subjects that the patients with anxiety regarding anesthesia would like to receive real information about, or the subjects those patients are anxious about. Thus, we think that it would be appropriate to explain these subjects carefully to the patients prior to the surgery.

Many studies used STAI test for the evaluation of anxiety, but we chose APAIS, because APAIS is much easy to use for both patient and the doctor, which have fewer questions, and it is as reliable as any other test. Studies investigated to evaluate anxiety with STAI or other tests, but they are hard to use for clinical routine, because they are not practical. APAIS is both reliable and practical [1]. It is a tool that has the capacity to differentiate between patient’s anxiety and desire for information. Anesthesiologists can enhance patients’ anesthetic experience, depending on the patients’ answers, like placing emphasis on management of perioperative time or just giving a much more detailed information about the operation that the patients are going to have [1]. Therefore, APAIS may be a useful tool for the routine use in the preoperative visits [1]. The point of our study that differentiates from other studies was additionally we asked the patients their anesthesia-related concerns and we lightened the association of anesthesia-related concerns and APAIS. It is necessary to intervene if patients show higher anxiety, because perioperative anxiety is associated with increased autonomic variations and increased anesthetic requirement and augmented postoperative pain [4–6]. As a result of these complications, many authors reported extended recovery period and the length of hospital stay [4]. Therefore, it is critical to reduce the anxiety of the patients. In the literature, there is plenty of evidence that demonstrates the preoperative information as playing a crucial role to diminish perioperative anxiety [23]. When an anesthesiologist detects patients with high anxiety, they should use audiovisuals, psychoeducational information, and preoperative nursing visits to reduce it. These interventions have shown to reduce anxiety and deliver supplementary information to the patients [23]. We can achieve improved results for patients when they have lower levels of anxiety.

The first limitation of our study is that it was a monocentric study. If the study could be performed in a multicentric manner, its efficacy, and the patient population, it included would have been higher. Another limitation was that we could not include patients hospitalized in the wards and only included the patients who came to our anesthesia clinic.

## Conclusion

In conclusion, we think that being aware of the patients’ anxiety and finding appropriate approaches for their anxieties can be valuable. APAIS is an effective method to measure patient anxiety and it might be beneficial to use during preoperative visits. Patient satisfaction and superior outcomes can be achieved in this way.

## Abbreviations

APAIS: Amsterdam Preoperative Anxiety and Information Scale
ASA: American Society of Anesthesiology
OR: odds ratio
SD: standard deviation
